# The Bradford Hill considerations on causality: a counterfactual perspective

**DOI:** 10.1186/1742-7622-2-11

**Published:** 2005-11-03

**Authors:** Michael Höfler

**Affiliations:** 1 Clinical Psychology and Epidemiology, Max Planck Institute of Psychiatry, Kraepelinstrasse 2-10, 80804 München, Germany

## Abstract

Bradford Hill's considerations published in 1965 had an enormous influence on attempts to separate causal from non-causal explanations of observed associations. These considerations were often applied as a checklist of criteria, although they were by no means intended to be used in this way by Hill himself. Hill, however, avoided defining explicitly what he meant by "causal effect".

This paper provides a fresh point of view on Hill's considerations from the perspective of counterfactual causality. I argue that counterfactual arguments strongly contribute to the question of when to apply the Hill considerations. Some of the considerations, however, involve many counterfactuals in a broader causal system, and their heuristic value decreases as the complexity of a system increases; the danger of misapplying them can be high. The impacts of these insights for study design and data analysis are discussed. The key analysis tool to assess the applicability of Hill's considerations is multiple bias modelling (Bayesian methods and Monte Carlo sensitivity analysis); these methods should be used much more frequently.

## Introduction

Sir Austin Bradford Hill (1897 – 1991) was an outstanding pioneer in medical statistics and epidemiology [1-4]. His summary of a lecture entitled "The environment and disease: Association or causation" [5] had an enormous impact on epidemiologists and medical researchers. Ironically, this paper became famous for something it was by no means intended to be [6,7]: a checklist for causal criteria (e.g. [8-10]).

Hill [5] provided nine considerations for assessing whether an observed association involved a causal component or not. These considerations were influenced by others before him [11,12]. He avoided defining explicitly what he meant by a causal effect, although seemingly he had the counterfactual conceptualisation mind. My core thesis in this paper is that counterfactual arguments contribute much to the question of when to apply Hill's considerations to a specific causal question. This is not to say that other conceptualisations of causality would not contribute to clarifying Hill's considerations, but the counterfactual model is the one that directly relates to many statistical methods [13,14], and it links the "metaphysical" side of causality to epidemiological practice. Moreover, I shall argue that some of Hill's considerations involve many counterfactuals in a broader causal system, and that the heuristic value of these considerations can be low.

### Analysis

#### Counterfactual causality

Hill [5] avoided defining exactly what he meant with a causal effect:

*"I have no wish, nor the skill to embark upon a philosophical discussion of the meaning of 'causation'*."

However, it seems that he applied the counterfactual model because he then writes:

"... *the decisive question is whether the frequency of the undesirable event B would be influenced by a change in the environmental feature A*."

Counterfactual causality dates back at least to the 18^th ^century Scottish philosopher David Hume [15] but only became standard in epidemiology from the 1980s. Being the inventor of randomised clinical trials [3,4], Hill was strongly influenced by the idea of randomised group assignment, which precludes confounding. The idea of randomisation, invented by R.A. Fisher's in the 1920s and 1930s was, in turn, stimulated by Hume [3]. As Fisher and Hill were friends for at least some years [3], it seems likely that Hill was strongly influenced by counterfactual thinking. Today, the counterfactual, or potential outcome, model of causality has become more or less standard in epidemiology, and it has been argued that counterfactual causality captures most aspects of causality in health sciences [13,14].

To define a counterfactual effect, imagine an individual *i *at a fixed time. Principally we assume that

(a) this individual could be assigned to both exposure levels we aim to compare (*X *= 0 and *X *= 1 respectively) and

(b) that the outcome *Y*_*i *_exists under both exposure levels (denoted by *Y*_*i0 *_and *Y*_*i1 *_respectively) [[14] and references therein].

The causal effect of *X *= 1 versus *X *= 0 within an individual *i *at the time of treatment or exposure assignment can be defined as [13-20]:

*Y*_*i1 *_*- Y*_*i0*_.

Note that the use of the difference measure is not exclusive – for strictly positive outcomes one can also use the ratio measure *Y*_*i1*_/*Y*_*i0*_. For a binary outcome, this definition means that the outcome event occurs under one exposure level but not under the other. Therefore, a causal effect of a binary event is a necessary condition without which the event would not have occurred; it is not necessarily a sufficient condition. Clearly, the outcome is not observable under at least one of the two exposure levels of interest. Thus, the outcome has to be estimated under the unobserved or *counterfactual *condition, known as the *counterfactual *or *potential *outcome.

According to Rothman [21], a comprehensive causal mechanism is defined as a set of factors that are jointly sufficient to induce a binary outcome event, and that are minimally sufficient; that is, under the omission of just one factor the outcome would change. Rothman [21] called this the *sufficient -component cause model*. A similar idea can be found in an earlier paper by Lewis [22]. Since several causal mechanisms are in line with the same specific counterfactual difference for a fixed individual at a fixed time, the sufficient-component cause model can be regarded as a finer version of the counterfactual model [[14], and references therein].

As there are often no objective criteria to determine individual counterfactual outcomes, the best option is usually to estimate population average effects. The population average effect is defined as the average of individual causal effects over all individuals in the target population on whom inference is to be made. The estimation of average causal effects in epidemiology is subject to various biases [23]. These biases are determined both by the study design and the mechanism that generates the data. In a randomised controlled trial (RCT), bias due to confounding cannot occur, but confounders might be distributed unequally across treatment levels by chance, especially in small samples. If compliance is perfect, there is no measurement error in the treatment. Other biases might still occur, however, such as bias due to measurement error in the outcome and selection bias (because individuals in the RCT might not represent all individuals in the target population). Observational studies are prone to all kinds of biases, and these depend on the causal mechanism underlying the data. For instance, bias due to confounding is determined by the factors that affect both exposure and outcome, and the distribution of these factors.

I shall demonstrate that most of Hill's considerations involve more than the *X *– *Y *association and biases in that association; their application depends on assumptions about a comprehensive causal system, of which the *X *– *Y *effect is just one component. I argue that the heuristic value of Hill's considerations converges to zero as the complexity of a causal system and the uncertainty about the true causal system increase.

#### The Bradford Hill considerations

The discussion of Hill's considerations is organised as follows: first, I use my own wording (in italics) to summarise the respective consideration. Hill's own argumentation is then briefly reviewed, followed by arguments of other authors (a subjective selection from the vast literature). I will then show which counterfactuals are involved in the application of a given consideration and what novel insights can be derived for the interpretation of study design and data analysis. To simplify the discussions, I will sometimes disregard random variation. Some of my arguments apply to several of Hill's considerations and I will occasionally not repeat them to avoid redundancy.

#### 1. Strength of association

##### A strong association is more likely to have a causal component than is a modest association

Hill [5] illustrated this point with the high risk ratios for the association between exposure levels of smoking and incidence of lung cancer. However, he demonstrated with two counter-examples that the absence of a strong association does not rule out a causal effect. Hill acknowledged that the impression of strength of association depended on the index used for the magnitude of association [5].

Rothman and Greenland [[18], p.24] provided counter-examples for strong but non-causal relationships. Note that, unlike ratio measures, difference measures tend to be small unless there is nearly a one-to-one association between exposure and outcome [24]. The fundamental problem with the choice of an effect measure is that "neither relative risk nor any other measure of association is a biologically consistent feature of an association... it is a characteristic of a study population that depends on the relative prevalence of other causes" [[18], p.24]. Rothman and Poole [25] described how studies should be designed to detect weak effects. For Rothman and Greenland [[18], p. 25] the benefit of the consideration on strength was that strong associations could not be solely due to small biases, whether through modest confounding or other sources of bias.

The consideration on strength involves two main counterfactual questions about biases that have presumably produced the observed association (in terms of a pre-specified index): how strong would the association be expected to be as compared to the observed association if the data were free of bias? Would the interval estimate that properly accounts for not only random, but also systematic error (uncertainty about bias parameters such as misclassification probabilities) allow for the desired conclusion or not? (A desired conclusion might be the simple existence of a causal effect or a causal effect of at least a certain magnitude, for instance a two-fold increase in risk.)

Bias can be addressed with multiple bias modelling. Contemporary methods for multiple bias modelling include Bayesian methods and Monte Carlo sensitivity analysis (MCSA, which can be modified to be approximatively interpretable in a Bayesian manner under certain conditions [27]). These methods address the uncertainties about bias parameters by assigning prior distributions to them. Although Hill pointed out that non-random error was often under-estimated, these methods were hardly available in his time. With Bayesian and MCSA methods one can assess whether the observed magnitude of association is sufficiently high to allow for a certain conclusion. This requires a bias model including assumptions on which kinds of biases exist, how biases act together and which priors should be used for them. However, if one uses a bias model that addresses understood bias, the inference could still be distorted by misunderstood or unknown bias. Moreover, one can calculate which *X *– *Y *association and random error values would have allowed for the desired conclusion. One may also ask which priors on bias parameters would, if applied, have allowed for the desired conclusion ("reverse Bayesian analysis") and assess the probability that these priors are not counterfactual.

Clearly, high uncertainty about bias parameters requires larger associations than modest uncertainty does. In studies that control for several sources of bias, modest associations might still be indicative of a causal effect, whereas in more error-prone designs more bias and higher non-random error has to be taken into account. However, specifying a bias model can be a difficult task if the knowledge about biases is also limited. This uncertainty then carries over to the uncertainty on applying the consideration on strength. Despite this, there seems to be no alternative to multiple bias modelling when assessing which magnitude of association is necessary for the desired conclusion.

#### 2. Consistency

##### A relationship is observed repeatedly

For Hill [5], the repeated observation of an association included "different persons, places, circumstances and time". The benefit of this rule was that consistently finding an association with different study designs (e.g. in both retrospective and prospective studies) reduced the probability that an association would be due to a "constant error or fallacy" in the same study design. On the other hand, he pointed out that shared flaws in different studies would tend to replicate the same wrong conclusion. Likewise, differing results in different investigations might indicate that some studies correctly showed a causal relationship, whereas others failed to identify it.

This point is explained by Rothman and Greenland [[18], p. 25]: causal agents might require that another condition was present; for instance, transfusion could lead to infection with the human immunodeficiency virus only if the virus was present. Now, according to the sufficient-component cause model [20,21], and as stated by Rothman and Greenland [[18], p. 25], whether and to what extent there is a causal effect on average depends on the prevalences of complementary causal factors.

Cox and Wermuth [[28], pp. 225] have added the consideration that an association that does not vary strongly across the values of intrinsic variables would be more likely to be causal. If an association were similar across individuals with different immutable properties, such as sex and birth date, the association would be more likely to have a stable substantive interpretation. Variables other than *X *and *Y *might change as a consequence of interventions among other factors in a comprehensive causal system. One should be careful when applying this guideline; effect heterogeneity depends on the choice of the effect measure. This choice should be based on a relevant substantive theory and on correspondence with the counterfactual and sufficient-component cause model (the latter two indicating that differences rather than ratios should be used); both may, however, contradict [29].

From the counterfactual perspective, the following questions arise when asking whether to apply the consideration on consistency:

a) If the causal effect was truly the same in all studies, would one expect to observe different associations in different studies (possibly involving different persons, places, circumstances and time)? To what degree would the associations be expected to differ?

b) If the causal effect varied across the studies, would one expect to observe equal or different associations? What magnitude of differences would one expect?

Note that in the presence of effect modifiers there exists no such thing as "the causal effect", the effect modifiers need to be fixed at suitable values. Also note that only a) or b) is actually counterfactual depending on whether the effect truly varies across the different studies or not. Answering these questions requires a comprehensive causal theory that indicates how different entities (individual factors, setting, time, etc.) act together in causing *Y*. Within such a causal system one can predict how the *X *– *Y *association should change if one used different persons, places, circumstances and times in different studies. As one can only observe associations this also involves bias, and bias might operate differently in different studies.

An observed pattern of association across the different studies that is in line with the expected pattern would provide evidence for an effect of *X *on *Y *if the underlying causal theory applies. Another pattern would indicate that there is either no effect of *X *on *Y *or that the supposed theory is false. In complex situations and bias-prone designs, the probability might be substantial that a causal theory does not include important features that change the expected *X *– *Y *association. Here, the uncertainty regarding whether or not to demand an association (or which magnitude of association) could be high, and so the consistency consideration might bring more harm than benefit.

#### 3. Specificity

##### A factor influences specifically a particular outcome or population

For Hill [5], if one observed an association that was specific for an outcome or group of individuals, this was a strong argument for a causal effect. In the absence of specificity, Hill alludes to fallacies in applying this rule to conclude the absence of a causal effect: Diseases may have more than one cause (which Hill considered to be the predominant case). In turn, a factor might cause several diseases. According to Hill, the value of this rule lay in its combination with the strength of an association: For instance, among smokers, the risk of death from lung cancer should be elevated to a higher degree as compared to the risk of other causes of death. Hill's consideration on specificity for persons apparently contradicts his consideration on consistency, where repeatedly observing an association in different populations would increase the evidence for a causal effect.

Rothman and Greenland [[18], p. 25] have argued that when applying this rule one assumes that a cause had one single effect. This assumption is often meaningless; for instance, smoking has effects on several diseases. They considered the demanding of specificity "useless and misleading". Cox and Wermuth [[28], p. 226f.] pointed out that this rule applies to systems where quite specific processes act, rather than to systems where the variables involved represent aggregates of many characteristics. Weiss [30] has mentioned a situation in which it can be meaningful to require specificity with respect to an outcome: a theory could predict that an exposure affects a certain outcome, but does not affect other particular outcomes. He illustrated this with the example that wearing helmets should be protective specifically against head injury, not against injury of other parts of the body. If wearing helmets also protected against other injuries, so he argues, this could be indicative that the association is confounded by more careful riders tending to use helmets. He provided similar arguments for specificity of exposure and specificity with regard to individuals in whom a theory predicts an effect.

Counterfactual causality, and the logically equivalent causal graphs [19,30], generalise the argument of Weiss [30] and solve the problem of specificity with respect to other exposures and outcomes: outcomes other than the one under consideration (*Y*) must be related with the exposure (*X*) if they are either part of the causal chain between *X *and *Y *or a causal consequence of *Y*. Otherwise, they must not be associated with *X*. In the example above, other injuries are neither part of the causal chain between wearing helmets and head injury, nor the causal consequence of head injury; the association between wearing helmets and head injury might share the common cause of carefulness if wearing helmets were related to both head and other injury. Likewise, exposures other than *X *must be associated with *Y *if they belong to the causal chain between *X *and *Y*. Whether exposures that occur before *X *are associated with *Y *or not is not informative about causality between *X *and *Y*.

When applying this consideration, a bias model is required for each association in the entire causal system, involving the assessment of as many counterfactual differences as there are associations. A single wrong conclusion about the existence of a particular effect might still yield a graph that contradicts the theory and, thus, a wrong conclusion about the existence of the *X *– *Y *effect. RCTs only allow a small number of factors to be simultaneously randomised, and whether they are associated with one another is just a question of how the randomisation is done. The assessment of specificity with respect to other factors in RCTs is therefore limited. Cohort studies are more useful here, but confounding and measurement error in the exposure are of higher importance. To conclude, the consideration of specificity appears to be useful only when a causal system is simple and the knowledge about it is largely certain.

#### 4. Temporality

##### The factor must precede the outcome it is assumed to affect

Hill [5] introduced this reflection with the proverb "Which is the cart and which is the horse?" For instance, he asked whether a particular diet triggered a certain disease or whether the disease led to subsequently altered dietary habits. According to Hill, temporal direction might be difficult to establish if a disease developed slowly and initial forms of disease were difficult to measure.

Considering individuals in whom *X *has occurred before *Y*, it is logically not possible that *X *would have changed if *Y *had changed, because *X *is fixed at the time when *Y *occurs (or not). Thus, *Y *cannot have caused *X *in these individuals. This is, indeed, the only sine qua non criterion for a counterfactual effect in a single individual [[7], p. 27, 11] – a point missed by Hill. Note that there is no logical link to individuals in whom *Y *has occurred before *X*. Among those, *Y *might or might not have caused *X *[[7], p. 25].

Even more confusion in applying this criterion arises when one aggregates information across several individuals. Some researchers believe that an association that is only observed in one direction is more likely to be causal than an association that is observed in both directions. If the presence of a certain disease (*X*) is associated with a higher subsequent incidence rate of another disease (*Y*), and if prior *Y *also predicts an elevated probability of subsequent onset of *X*, it is sometimes assumed that a shared vulnerability was the common cause of both associations. This possibility has, for instance, been discussed for the role of anxiety in the development of depression [31].

Applying this argument, however, requires a causal system that produces no *Y *– *X *association. This requires sufficient knowledge about shared risk factors of *X *and *Y*. To assess temporal order between *X *and *Y*, a longitudinal design is preferable to a design in which the temporal direction between *X *and *Y *has to be assessed retrospectively. In RCTs, temporal direction can be established without error.

#### 5. Biological gradient

##### The outcome increases monotonically with increasing dose of exposure or according to a function predicted by a substantive theory

Hill [5] favoured linear relationships between exposure level and outcome, for instance, between the number of cigarettes smoked per day and the death rate from cancer. If the shape of the dose-response relationship were a more complex, especially a non-monotonic, function, this would require a more complex substantive explanation.

Others have been less demanding and more specific in their definition of a dose-response relation, requiring only a particular shape of relationship (not necessarily linear or monotonic), which is predicted from a substantive theory [[28], p. 225]. Rothman and Greenland [[18], p. 26] have argued that parts of J-shaped dose-response curves might be caused by the respective exposure levels while others might be due to confounding only. They also provided a counter-example for a non-causal dose-response association. To demand a dose-response relationship could be misleading if such an assumption contradicted substantive knowledge. No dose-response relationship in presumably causal effects has been found, for example, between the intake of inhaled corticosteroids and lung function among asthma patients [33] or in the pharmacotherapy of mental disorders [34]. Further examples and similar arguments as above had been provided previously by Lanes and Poole [35].

Counterfactual causality defines the difference between each pair of exposure levels as a distinct causal effect. The consideration on biological gradient is therefore again not a consideration on a specific causal difference but a consideration on a broader causal system involving several exposure levels. It requires a substantive theory that predicts how the outcome should change when the exposure varies over several levels. If there are *k *exposure levels, then this theory has to predict *k-1 *counterfactual differences. Some theories demand a gradient over the levels and others do not, while different theories might demand different gradients.

When applying this consideration, bias has to be properly corrected for each of the *k-1 *observed associations. If the observed sequence of associations over the exposure levels is in line with a theory and bias is properly addressed for each comparison, this provides evidence for the theory; otherwise, the theory, or at least one bias model, is false. Here, causal differences between specific exposure levels might in any case exist.

Several exposure levels are required to establish a dose-reponse relationship. On the other hand, the more exposure levels there are, the higher is the danger of mis-applying this consideration, because a single wrong conclusion (among *k-1 *possible wrong conclusions) about the existence of a specific causal difference might be sufficient for a wrong conclusion on the overall theory. RCTs are particularly useful for assessing dose-response relationships, because they avoid some biases that are sometimes difficult to correct for using other study designs.

#### 6. Plausibility

##### The observed association can be plausibly explained by substantive matter (e.g. biological) explanations

For Hill [5], the presence of a biological explanation supported the drawing of a causal conclusion. On the other hand, in the absence of such a theory "the association we observe may be one new to science or medicine and we must not dismiss it too light-heartedly as just too odd" [5].

Cox and Wermuth [[28], p. 226] have added the extremely important point that a relationship that is predicted prospectively is much more convincing than one that is provided retrospectively; after observing an association, it is often easy to give a plausible explanation. According to Rothman and Greenland [[18], p. 26], the assessment of plausibility is subject to the prior beliefs of individual researchers. The weight of these prior beliefs could be balanced against the weight of the observed association in Bayesian inference. However, Bayesian analysis is not able to "transform plausibility into a causal criterion".

Based on the arguments of these authors there are two relevant counterfactual questions that researchers should ask themselves; they are related to plausibility, although they are not sufficient to clarify this consideration on their own:

a) If the observed association is in line with substantive knowledge, would you have assigned it a lower weight (relatively to the weight of substantive knowledge) if your observations had not been in line with substantive knowledge?

b) If the observed association is not in line with substantive knowledge, would you have assigned it a higher weight (relatively to the weight of substantive knowledge) if your observations had been in line with substantive knowledge?

Only if researchers are able to answer a) or b) (respectively) honestly with "yes" can one assume that the application of this consideration did not depend on the study results. Clearly, one cannot even be 100% confident that an answer is really honest for someone's own thoughts. The danger in applying this consideration is twofold: researchers might assign a higher weight to substantive knowledge if it agrees with their own prior opinion and might assign a lower weight otherwise. Substantive knowledge might be inconsistent and conflicting information could be weighted according to someone's assessment of the accuracy of the information. Likewise, scientists might choose the substantive knowledge they apply according to whether it fits to their prior opinions. The question remains how to weight prior opinions relatively to the observed results. This involves many design issues, such as the sample sizes in the present data and in other data, and the questions of how bias acted and was addressed in different studies.

#### 7. Coherence

##### A causal conclusion should not fundamentally contradict present substantive knowledge

Hill [5] used the term "generally known facts" to indicate that the knowledge against which an association is evaluated has to be undisputable. Laboratory evidence that is in line with an association would underline a causal conclusion and help to identify the causal agent. Again, the absence of such knowledge would not be indicative of a non-causal explanation.

The difference in Hill's definitions of plausibility and coherence appears to be subtle [[7], p. 25]. Whereas plausibility is worded positively (an association that should be in line with substantive knowledge), coherence is verbalised negatively (an association that should not conflict with substantive knowledge). Rothman and Greenland [[7], p. 25] have drawn attention to the possibility that such conflicting knowledge might itself be wrong. Susser [11] has tried to retain this consideration by defining different subclasses of coherence depending on where knowledge comes from.

A subtle difference between coherence and plausibility is that plausibility asks: "Could you imagine a mechanism that, if it had truly operated (which could be counterfactual), would have produced results such as those observed in the data?" By contrast, coherence asks: "If you assume that the established theory is correct (i.e. not counterfactual), would the observed results fit into that theory?" Whereas the consideration of coherence would reject the observed result to be non-causal if it contradicted a predominant theory, plausibility leaves the researcher more room regarding which particular piece of substantive knowledge to evaluate the results against.

#### 8. Experiment

##### Causation is more likely if evidence is based on randomised experiments

Hill [5] argued that a causal interpretation of an association from a non-experimental study was supported if a randomised prevention derived from the association confirmed the finding. For instance, after finding that certain events were related to the number of people smoking, one might forbid smoking to see whether the frequency of the events decrease consecutively.

To Rothman and Greenland [[7], p. 27], it has not been clear whether Hill meant evidence from animal or human experiments. Human experiments were hardly available in epidemiology, and results from animal experiments could not easily be applied to human beings. To Susser [11], Hill's examples suggested that he meant intervention and active change rather than research design. Both Susser [11] and Rothman and Greenland [[7], p. 27] stated that results from randomised experiments provided stronger evidence than results based on other study designs, but always had several possible explanations. Cox and Wermuth [[28], p. 225f.] relaxed that criterion by replacing the qualitative difference between experimental and non-experimental studies with the rather quantitative conception of "strength of intervention": an observed difference would be more likely to be causal if it followed a massive intervention. This is motivated by the possibility that a change following a modest intervention could result from the circumstances of a treatment rather than from the treatment itself. One might add that the Cox and Wermuth consideration requires a modest intervention to be precluded from having a strong influence – an assumption that is certainly context-dependent to a high degree.

In terms of counterfactual causality, the distinction between massive and modest interventions is irrelevant, because a causal effect is only defined for a fixed index and a fixed reference condition. Hence, if interpreted in terms of strength of intervention, this is again not a consideration on a specific causal difference, but rather a consideration on a comprehensive causal theory (as the one on biological gradient). Such a theory is required in order to decide what is a modest and what is a strong intervention.

If the consideration on experiment is interpreted in terms of avoiding some biases in estimating a specific causal effect by conducting an RCT, it should be generalised as follows: observed associations should equal the true counterfactual difference as closely as possible (despite random error). Bias is reduced either by using a study design that avoids major biases or by properly correcting for bias. Clearly, avoiding bias is preferable to correcting for it, but it is often impossible to avoid some biases. As already mentioned, in RCTs with perfect compliance, confounding cannot occur (although confounders might be distributed unequally by chance) and there is no measurement error in the exposure. However, bias due to measurement error could still occur in the outcome, and there may be bias due to selection, missing data, etc. [22]. Thus, Hill's original formulation [5] covered only one or two among a variety of possible biases.

Instead, two more general question arise: which study design is likely to validly identify a presumed causal effect? And, if the optimal study design is not possible, how can bias be accurately corrected for? As in the consideration on strength, this can be summarised by: which results would be expected to be observed if the data were free of any bias? A causal effect is more likely if, after bias adjustment, the interval estimate excludes the null value, and it is even more likely if the lower boundary is far from the null value. If adjustment is done properly, systematic error in the corrected interval estimate decreases if the knowledge about biases increases. As a consequence, one can hardly ever demonstrate a causal effect if biases are poorly understood – this is the case even in large samples, because the associated systematic error in the results would remain even while random error decreases.

#### 9. Analogy

##### For analogous exposures and outcomes an effect has already been shown

Hill [5] wrote that it would be sometimes acceptable to "judge by analogy". He gives the following example:

*"With the effects of thalamoide and rubella before us we would surely be ready to accept slighter but similar evidence with another drug or another viral disease in pregnancy*."

Susser [11] interpreted Hill as someone claiming that "when one class of causal agents is known to have produced an effect, the standards for evidence that another agent of that class produces a similar effect can be reduced." Rothman and Greenland [[18], p. 27] retorted:

*"Whatever insight might be derived from analogy is handicapped by the inventive imagination of scientists who can find analogies everywhere. At best, analogy provides a source of more elaborate hypothesis about the associations under study; absence of such analogies only reflects lack of imagination or lack of evidence*."

When applying the consideration on analogy scientists should ask themselves: would you expect the same association if you used settings analogous to those in other studies (while taking biases into account, which may differ across the studies)? The term "analogous" suggests that the entities in external studies are only similar to those in the observed data (but not identical). This requires an additional modelling of the counterfactual effects of using analogous but not identical entities in different studies. This makes the application of the analogy consideration even more uncertain than the application of considerations on plausibility and coherence.

## Conclusion

Hill himself used the terms "viewpoints" and "features to be considered" when evaluating an association. His aim was to unravel the question: "What aspects of this association should we especially consider before deciding that the most likely interpretation of it is causation?"[5] He expressed his ambivalence about the usefulness of his own considerations as follows:

*"None of my nine viewpoints can bring indisputable evidence for or against the cause-and-effect hypothesis*..."

Rothman and Greenland's conclusion [36] suggests that there are no causal criteria at all in epidemiology :

*"Causal inference in epidemiology is better viewed as an exercise in measurement of an effect rather than as criterion-guided process for deciding whether an effect is present or not*."

The usefulness of the counterfactual approximation of Hill's considerations is that their heuristic value can be assessed by answering counterfactual questions. I have argued that the application of seven of the nine considerations (consistency, specificity, temporality, biological gradient, plausibility, coherence and analogy) involves comprehensive causal theories. Complex causal systems comprise many counterfactuals and assumptions about biases. If complexity becomes very large, the uncertainty regarding whether or not to apply a given consideration can be expected to approach a decision made by coin toss. Thus, with increasing complexity, the heuristic value of Hill's considerations diminishes.

Here, an original argument of Hill [5] becomes of particularly important: the required amount of evidence for a causal effect should depend on the possible consequences of interventions derived from causal conclusions. If a causal conclusion needed an action that brought about more harm if wrongly taken than benefit if rightly taken, a correspondingly high amount of evidence would be required. If the relationship between benefit and harm were converse, less evidence would be necessary. The major tool to assess the applicability of these considerations is multiple bias modelling. Multiple bias models should be much more frequently used. Moreover, the decision as to whether or not to apply one of these considerations is always implicitly based on one or several multiple bias models. For instance, demanding an association of at least a certain magnitude is logically equivalent to the "true bias model" being part of the set of multiple bias models in which priors on bias parameters would require at least this magnitude of association to be observed.

One may ask the counterfactual question of how epidemiology and medical research would have developped if Hill had been more explicit in recommending when to apply each of his considerations. I am far from claiming to be able to answer this question, but I consider my speculation being worth mentioning. In their paper entitled "The missed lessons of Sir Austin Bradford Hill" [6] Phillips and Goodman reviewed malpractices denounced by Hill that were still being made later in practice: over-emphasis of statistical tests, systematic error being under-estimated and cost/benefit trade-offs being disregarded in intervention decisions. Hill's considerations were misused as "causal criteria", and they were taught more often than more sound causal conceptions [6]. There is no reason to believe that more explicit recommendations on when to apply his considerations would have been better heeded; the cautionary notes that Hill actually made were largely ignored.

My own experience is that scientific recommendations are widely followed if they provide easy guidance; recommendations that call for complex action are frequently ignored. My guess is that this is due to many researchers' desire for simple and globally applicable answers. This desire leads to misinterpretation of scientific texts and to taking individual statements out of their context. More pessimistically, the question of which guidance is followed depends on which guidelines are in line with the desired answer. Therefore, it seems likely that, even if Hill's paper had not been published, scientists' desire for simple answers would have caused another paper to be written or to be misinterpreted in the same way as happened with Hill's [5] article.

## List of abbreviations

MCSA: Monte Carlo sensitivity analyses

RCT: randomised and controlled trial

## Competing interests

The author(s) declare that they have no competing interests.
